# The effects of feeding resistant starch on apparent total tract macronutrient digestibility, faecal characteristics and faecal fermentative end-products in healthy adult dogs*

**DOI:** 10.1017/jns.2014.28

**Published:** 2014-09-30

**Authors:** Alison N. Beloshapka, Lucille G. Alexander, Preston R. Buff, Kelly S. Swanson

**Affiliations:** 1 Department of Animal Sciences, University of Illinois, Urbana, IL, USA; 2 WALTHAM^®^ Centre for Pet Nutrition, Leicestershire, UK; 3 The Nutro Company, Franklin, TN, USA; 4 Division of Nutritional Sciences, University of Illinois, Urbana, IL, USA

**Keywords:** Canine nutrition, Whole grains, Nutrient digestibility, Biscuits, Treats, Resistant starch, RS, resistant starch

## Abstract

The benefits of whole grain consumption have been studied in human subjects, but little research exists on their effects in dogs. The objective of the present study was to test the effects of resistant starch (RS) in the diet of healthy adult dogs. Twelve adult Miniature Schnauzer dogs (eight males, four females; mean age: 3·3 (1·6) years; mean body weight: 8·4 (1·2) kg; mean body condition score: D/ideal) were randomly allotted to one of three treatment groups, which consisted of different amounts of RS supplied in a biscuit format. Dogs received either 0, 10 or 20 g biscuits per d (estimated to be 0, 2·5 or 5 g RS per d) that were fed within their daily energetic allowance. A balanced Latin square design was used, with each treatment period lasting 21 d (days 0–17 adaptation; days 18–21 fresh and total faecal collection). All dogs were fed the same diet to maintain body weight throughout the study. Dogs fed 5 g RS per d had lower (*P* = 0·03) fat digestibility than dogs fed 0 gRS per d, but DM, organic matter and crude protein digestibilities were not affected. Faecal fermentative end-products, including SCFA and branched-chain fatty acids, ammonia, phenols and indoles, and microbial populations were not affected. The minor changes observed in the present study suggest the RS doses provided to the dogs were too low. Further work is required to assess the dose of RS required to affect gut health.

A healthy gastrointestinal tract is important for the overall health of dogs. It is well known that diet can alter gastrointestinal health. The benefits of consuming whole grains have been greatly studied in human subjects^(^^1^^–^^3^^)^, but little research exists on their effects in dogs. Previous research has demonstrated the benefits of feeding dietary fibre and prebiotics to dogs^(^^4^^–^^7^^)^. Similarly, resistant starch (RS) is highly fermentable and can modulate the gut microbial composition and faecal SCFA concentrations^(^^8^^)^. Feeding whole grains, which contain RS when minimally processed^(^^9^^,^^10^^)^, may have similar beneficial effects on the canine gut.

As extrusion conditions can alter the chemical composition of ingredients^(^^9^^)^, the use of a treat matrix produced by baking may be a more effective way to deliver the RS portion of whole grains to the colon of a dog. A treat matrix will allow for more whole grains to be included, without the need for fine grinding and complete gelatinisation that occurs with extrusion. The objective of the present study was to test the effects of RS in healthy adult dogs by feeding a biscuit treat, containing whole brown rice, whole wheat and whole grain oatmeal. The aim of this trial was to determine a RS dose (0, 2·5 or 5 g RS per d) that manipulates faecal bifidobacteria, lactobacilli and fermentative end-products, but that does not negatively affect stool quality in healthy dogs fed treats.

## Materials and methods

### Animals and diets

The present study was performed at the WALTHAM^®^ Centre for Pet Nutrition and all animal care and study procedures were approved by the Animal Welfare and Ethics Review Body. A total of twelve healthy adult Miniature Schnauzer dogs (eight males; four females; mean age: 3·3 (1·6) years; mean body weight: 8·4 (1·2) kg; mean body condition score: D/ideal) were included in the study. A balanced 3 × 3 Latin square design with 21 d trial periods was conducted. Each period consisted of a diet adaptation phase (days 0–17) and a total and fresh (within 15 min of defecation) faecal collection phase (days 18–21). During the adaptation phase, dogs were pair-housed overnight, with free access to indoor and outdoor paddock areas, where they were also group-housed during the day. During the collection phase, dogs were individually housed overnight and group-housed during the day.

All dogs were fed a complete and balanced canned diet twice daily (08:30 and 15:00) to maintain body weight throughout the study. The diet consisted of 92·4 g protein/4184 kJ (1000 kcal) metabolisable energy and 65·0 g fat/4184 kJ (1000 kcal) metabolisable energy. Intake for each dog was recorded at each meal. Prior to receiving their meals, dogs were given 0, 10 or 20 g of biscuit treats per d (split into two doses) that provided approximately 0, 2·5 or 5 g RS/d from the biscuits. The biscuits consisted of 43·2 g protein/4184 kJ (1000 kcal) metabolisable energy and 33·5 g fat/4184 kJ (1000 kcal) metabolisable energy. Dogs were weighed and assessed for body condition score^(^^11^^)^ weekly. The chemical composition of the maintenance diet and biscuits can be found in Table 1.

**Table 1.** Chemical composition of maintenance diet and biscuits

	Diet	
Chemical composition	Maintenance diet	Biscuits
DM (%)	20·30	95·06
	% DM basis	
Organic matter	84·39	96·95
Crude protein	36·13	15·74
Acid hydrolysed fat	25·41	12·21
Total dietary fibre	8·86	10·47
Insoluble	4·40	7·44
Soluble	4·46	3·02
Gross energy (kJ/g)	22·48	20·72
ME_AAFCO_* (kJ/g)	16·38	15·22
Free glucose (FG)	0·18	0·17
Total starch	16·11	58·20
Total starch (without FG)	15·95	58·05
Digestible starch	12·77	46·46
Digestible starch (without FG)	12·61	46·31

AAFCO, Association of American Feed Control Officials.

*Metabolisable energy (ME)_AAFCO_=35·564 kJ ME/g fat + 14·644 kJ ME/g CP + 14·644 kJ ME/g nitrogen-free extract.

### Sample collection

During the 4-d collection phase, all faecal output was collected, weighed, scored and frozen at −20°C for further analysis; one fresh faecal sample from each dog per period was also collected. Faecal quality was assessed on each sample according to visual appearance as described by Rolfe *et al.*^(^^12^^)^ with grade 1 representing ‘hard, dry, crumbly faeces’ and grade 5 ‘watery diarrhoea’. To account for all the discernible points in between, half-scores are used, giving a total of nine possible categories, with a score of 1·5–2·5 considered ideal.

The fresh faecal sample was collected within 15 min of defecation on day 1 of the 4-d collection phase and prepared immediately to minimise loss of volatile components. Samples were weighed and pH measured using a Mettler Toledo FG2/EL2pH meter equipped with a Mettler Toledo LE 438 probe (Mettler Toledo) after mixing 2 g of fresh faeces into 18 ml of sterile water. Fresh faecal DM was determined (105°C). Aliquots of faeces for analysis of phenols and indoles were frozen at −20°C immediately after collection. An aliquot (5 g) of faeces was mixed with 5 ml 2 m HCl for ammonia, SCFA and branched-chain fatty acid determination and stored at −20°C until analysed. Aliquots of fresh faeces were transferred to sterile cryogenic vials (Nalgene) and frozen at −80°C until DNA extraction for microbial analysis.

### Chemical analyses

Dried diet and faecal samples were ground through a 2-mm screen in a Wiley Mill (intermediate, Thomas Scientific). Samples were analysed according to procedures by the Association of Official Analytical Chemists (AOAC) for DM (105°C), organic matter and ash^(^^13^^)^. Crude protein content was calculated from Leco total N values (TruMac^®^ N, Leco Corporation)^(^^13^^)^. Total lipid content (acid hydrolysed fat) of the samples was determined according to the methods of the American Association of Cereal Chemists (AACC)^(^^14^^)^ and Budde^(^^15^^)^. Gross energy of the samples was measured using an oxygen bomb calorimeter (model 1261, Parr Instruments). Dietary fibre concentrations (total dietary fibre, soluble dietary fibre and insoluble dietary fibre) were determined according to the method of Prosky *et al.*^(^^16^^)^.

SCFA and branched-chain fatty acid concentrations were determined by GC according to the method of Erwin *et al.*^(^^17^^)^ using a Hewlett-Packard 5890A series II gas chromatograph (Palo Alto) and a glass column (180 cm × 4 mm i.d.) packed with 10 % SP™-1200/1 % H_3_PO_4_ on 80/100+ mesh Chromosorb WAW (Supelco Inc.). Phenol and indole concentrations were determined using GC according to the method of Flickinger *et al.*^(^^7^^)^. Ammonia concentrations were determined according to the method of Chaney & Marbach^(^^18^^)^.

### Starch

Biscuit and diet subsamples were ground through a 0·5-mm screen in a Wiley Mill (intermediate, Thomas Scientific). The method of Muir & O'Dea^(^^19^^,^^20^^)^ was used to determine the amount of starch digested in the stomach and small intestine by measuring glucose in the supernatant resulting from acid–enzyme digestion of the substrate. Briefly, 0·2 g of each substrate was weighed in duplicate and exposed to pepsin/HCl, amyloglucosidase and α-amylase digestion. Tubes containing reagents but no substrate were run as blanks. All tubes were incubated for 15 h at 37°C and then centrifuged for 15 min. Glucose concentrations in the supernatant were determined by reading the absorbance of individual samples at 450 nm on a DU 640 spectrophotometer (Beckman Instruments) and comparing those values against a glucose standard curve. Digestible starch was determined by subtracting (free glucose × 0·9) from (total glucose/original sample weight) present in the supernatant after 15 h of digestion. Because the measurement of glucose was used to determine starch content, a correction factor of 0·9 for the difference in weight between a free glucose unit and a glucose residue in starch was used. Total starch content of samples was determined using the method of Thivend *et al.*^(^^21^^)^ with amyloglucosidase. RS was calculated by subtracting (digestible starch +(free glucose × 0·9)) from total starch. The released glucose value corresponds to the amount of glucose resulting from hydrolytic starch digestion that is available for absorption *in vivo*.

### Microbial analyses

Faecal DNA was extracted from freshly collected samples that had been stored at −80°C until analysis, using the PowerLyzer™ PowerSoil^®^ DNA Isolation Kit (MO BIO Laboratories, Inc.) according to the manufacturer's instructions. Extracted DNA was quantified using a Qubit^®^ 2·0 Fluorometer (Life Technologies™, Invitrogen™). Quantitative PCR was performed using specific primers for *Bifidobacterium* spp.^(^^22^^)^, *Lactobacillus* spp.^(^^23^^)^, *Escherichia coli*^(^^24^^)^ and *Clostridium perfringens*^(^^25^^)^. While *Bifidobacterium* and *Lactobacillus* are generally considered to be ‘beneficial’ microbes, *E. coli* and *C. perfringens* represent potential pathogens, and are commonly measured in prebiotic studies, thus, were also analysed for the present study. Amplification was performed according to DePlancke *et al.*^(^^26^^)^. Briefly, a 10-μl final volume contained 5 µl of 2 × SYBR Green PCR Master Mix (Applied Biosystems), 0·05 µl of bovine serum albumin, 15 pmol of the forward and reverse primers for the bacterium of interest, and 10 ng of extracted faecal DNA. Standard curves were obtained by harvesting pure cultures of the bacterium of interest in the log growth phase in triplicate, followed by serial dilution. Bacterial DNA was extracted from each dilution using a DNA extraction kit (Qiagen) and amplified with the faecal DNA to create triplicate standard curves (ABI PRISM 7900HT Sequence Detection System, Applied Biosystems). Colony-forming units in each dilution were determined by plating on specific agars; lactobacilli MRS (Difco, BD) for *Lactobacillus*, reinforced clostridial medium (*Bifidobacterium*, *C. perfringens*) and Luria Bertani medium (*E. coli*). The calculated log colony-forming units per ml of each serial dilution were plotted against the cycle threshold to create a linear equation to calculate colony-forming units per gram of dry faeces. Although the standard curves are meant to represent a group of bacteria, our quantitative PCR assays were based on a single bacterial strain within each group. As operon copy number is different among strains, a potential bias does exist in our assay, to minimise this each dog acted as its own control.

### Calculations and statistical analysis

Apparent total tract energy and apparent macronutrient digestibility values were calculated using the following equation: nutrient intake (g DM/d) − nutrient output (g DM/d)/nutrient intake (g DM/d) × 100. Data were analysed using the MIXED procedure of SAS (version 9·3, SAS Institute Inc.). Faecal score data were compared using the GLIMMIX procedure of SAS. The statistical model included period and dog as random effects, whereas treatment was a fixed effect. Data were analysed using the type 3 test of the MIXED procedure. All treatment least-squares means were compared using preplanned contrasts that tested for linear effects of RS supplementation. Means were separated using a protected least-squares difference with a Tukey's adjustment. Data were analysed using the UNIVARIATE procedure to produce a normal probability plot based on residual data and visual inspection of the raw data. A probability of *P* ≤ 0·05 was accepted as being statistically significant and *P* ≤ 0·10 accepted as trends.

## Results

All results can be found in Table 2. Dietary organic matter intake was greater (*P* = 0·05) in dogs fed 20 g biscuits per d compared with those fed 0 g biscuits per d (Table 2). Dogs consumed an average of 5·5 g RS per d when fed the canned diet alone. Therefore dogs fed 0, 10 or 20 g of biscuits per d consumed approximately 5·5, 8·0 or 10·5 g RS per d, respectively. Dogs fed 20 g biscuits per d had lower (*P* = 0·03) fat digestibility than dogs fed 0 g biscuits per d. DM, organic matter and crude protein digestibilities; faecal output, faecal DM% and faecal scores; faecal SCFA, branched-chain fatty acid and ammonia concentrations; and faecal microbiota were not affected.

**Table 2.** Food intake, faecal characteristics, apparent total tract macronutrient digestibility, faecal fermentative end-product concentrations and faecal microbial populations of adult dogs fed 0, 10 or 20 g biscuits per d (*n* 12)

	Treatment (g biscuits per d)				
	
Item	0	10	20	sem	*P* value Treatment
Food intake					
g DM per d	174·29	181·35	193·33	20·166	0·09
g OM per d	147·09^a^	154·31^ab^	165·60^b^	17·019	0·05
g CP per d	62·97	63·47	65·87	7·286	0·59
g fat per d	44·29	44·75	46·55	5·124	0·53
Gross energy (kJ/d)	3918·68	4059·71	4312·37	453·23	0·12
ME_C_* (kJ/g)	17·86	17·97	17·71	0·177	0·30
Faecal output (g/d) (as is)	142·19	135·66	151·02	21·214	0·41
Faecal output (g/d) (DMB)	35·47	34·81	38·17	4·33	0·35
Faecal output (as is)/food intake (DMB)	0·81	0·73	0·77	0·047	0·13
Digestibility					
DM, (%)	79·49	80·91	80·2	1·013	0·26
OM (%)	84·31	85·38	84·59	0·808	0·29
CP (%)	81·82	82·28	80·74	1·045	0·14
AHF (%)	95·02^b^	94·83^ab^	94·31^a^	0·506	0·03
Energy (%)	86·32	87·01	85·85	0·868	0·38
Faecal scores^†^	2·54	2·53	2·6	0·072	0·22
Faecal DM (%)	26·89	26·18	26·6	0·926	0·67
Faecal pH	6·81	6·72	6·41	0·196	0·25
Ammonia (μmol/g) DM	1035·41	1063·28	1157·85	735·53	0·66
SCFA (μmol/g DM)					
Acetate	247·44	270·66	274·73	23·695	0·38
Propionate	164·85	120·37	132·62	27·658	0·50
Butyrate	41·43	48·86	45·73	4·805	0·11
Total SCFA	453·17	440·45	453·07	41·809	0·96
Acetate/total SCFA	0·58	0·62	0·61	0·023	0·22
Propionate/total SCFA	0·33	0·27	0·29	0·024	0·13
Butyrate/total SCFA	0·10	0·11	0·10	0·006	0·21
BCFA (μmol/g DM)					
Valerate	0·70	0·80	0·92	0·101	0·15
Isovalerate	14·67	16·04	15·36	1·394	0·27
Isobutyrate	9·73	10·59	10·19	0·925	0·27
Total BCFA	25·10	27·43	26·47	2·392	0·26
Phenols and indoles (μmol/g DM)					
Phenol	1·07	1·23	1·45	0·412	0·42
Indole	2·78	2·86	2·93	0·411	0·91
Total phenols and indoles	3·85	4·09	4·35	0·717	0·63
Microbial population (CFU^‡^, log 10/g faecal DM)				
*Escherichia coli*	9·42	9·11	9·69	0·539	0·67
*Lactobacillus* spp.	9·87	9·98	9·66	0·563	0·76
*Bifidobacterium* spp.	7·01	6·88	6·74	0·269	0·39
*Clostridium perfringens*	9·64	9·74	9·65	0·653	0·97

OM, organic matter; CP, crude protein; DMB, DM basis; AHF, acid hydrolysed fat; BCFA, branched-chain fatty acids.

^a,b^Means not sharing a common superscript differ (*P* ≤ 0·05).

*ME_Calculated_ = [GE intake (kJ/d) − faecal GE (kJ/d) − [(CP intake/100) − (faecal CP/100)] × 1·25]/DM intake (g/d).

^†^Faecal score scale: 1 = hard, dry pellets; 2 = dry, well-formed stool; 3 = soft, moist, formed stool; 4 = soft, unformed stool; 5 = watery, liquid that can be poured.

^‡^Colony-forming units.

## Discussion

Even though RS has been demonstrated to affect faecal characteristics (e.g. faecal SCFA concentrations and gut microbial populations) in previous studies^(^^27^^,^^28^^)^, very little change was observed in this study. While fat digestibility decreased from 95·0 to 94·3 % and reached statistical significance, this change is of little biological relevance from an energetics standpoint. It is speculated that the limitation of treat intake (10 % of daily ME) and the minor changes observed in the present study suggest the RS/fermentable fibre doses provided by the whole-grain treats were too low. Additionally, the fermentable fibre included in the base diet may have interfered with the possible benefits of consuming RS from whole grains. At 10 % RS inclusion, Rideout *et al.*^(^^29^^)^ observed higher caecal butyric acid concentrations and lower caecal indole, isobutyric acid and isovaleric acid concentrations in pigs than control fed pigs. Additionally, Tachon *et al.*^(^^30^^)^ observed higher proportions of *Lactobacillus* and *Bifidobacterium* in mice-fed diets containing 18 % RS from Hi-maize 260^®^ than control-fed mice. As Hi-maize 260^®^ is composed of 60 % RS and 40 % DS, these diets contained approximately 10·8 % RS. Based on our food intake data of the dogs in the present study, they consumed approximately 1·4 and 2·7 % RS from the treats. Further studies are required to assess the effectiveness of higher doses of RS/fermentable fibre on macronutrient, faecal characteristics and fermentative end-products in the dog.
